# The role of water bridge on gas adsorption and transportation mechanisms in organic shale

**DOI:** 10.1038/s41598-024-66055-4

**Published:** 2024-07-01

**Authors:** Binhui Li, Yong Liu, Yubo Lan, Jiawei Li, Yue Lang, Sheikh S. Rahman

**Affiliations:** 1 State Key Laboratory of Continental Shale Oil, Daqing, 163712 China; 2 Daqing Oilfield Exploration and Development Research Institute, Daqing, 163712 Heilongjiang China; 3 School of Minerals and Energy Resources Engineering, UNSW, Sydney, 2052 Australia

**Keywords:** Saline water, Gas transportation, CCUS, Shale gas, Molecular dynamics, Environmental sciences, Energy science and technology, Nanoscience and technology

## Abstract

This work introduces and discusses the impacts of the water bridge on gas adsorption and diffusion behaviors in a shale gas-bearing formation. The density distribution of the water bridge has been analyzed in micropores and meso-slit by molecular dynamics. Na^+^ and Cl^−^ have been introduced into the system to mimic a practical encroachment environment and compared with pure water to probe the deviation in water bridge distribution. Additionally, practical subsurface scenarios, including pressure and temperature, are examined to reveal the effects on gas adsorption and diffusion properties, determining the shale gas transportation in realistic shale formation. The outcomes suggest carbon dioxide (CO_2_) usually has higher adsorption than methane (CH_4_) with a water bridge. Increasing temperature hinders gas adsorption, density distribution decreases in all directions. Increasing pressure facilitates gas adsorption, particularly as a bulk phase in the meso-slit, whereas it restricts gas diffusion by enhancing the interaction strength between gas and shale. Furthermore, ions make the water bridge distributes more unity and shifts to the slit center, impeding gas adsorption onto shale while encouraging gas diffusion. This study provides updated guidelines for gas adsorption and transportation characteristics and supports the fundamental understanding of industrial shale gas exploration and transportation.

## Introduction

Unconventional gas reservoirs, including shale and coalbed reservoirs, have drawn much attention these years, attributed to the high demand for natural fossil resources and the clean characteristics of unconventional reservoirs^1–3^. However, shale reservoirs are typically associated with connate water, the high content of which usually forms a water bridge that significantly impacts gas adsorption, transportation, and recovery processes^4–7^. Hence, understanding the effect of water bridges in shale reservoirs is crucial and essential for the industrial exploration of shale gas, estimation of in-place reserves, and CO_2_ sequestration potential. Therefore, this work focuses on the gas adsorption and transportation behaviors in the encroached subsurface environment, which has been studied at a shallow depth and needs to be noticed or addressed in previous studies, and reveals the water bridge’s impacting mechanisms behind the gas movement.

In shale gas-bearing gas reservoirs, the pore size ranges from angstrom to micrometer, with micro and mesopores comprising the prominent pores that possess primary gas adsorption capacity. To observe the mechanisms of water bridge impacts on gas adsorption in small-scale pores is a great challenge in the laboratory. Therefore, molecular dynamics (MD) simulation has emerged as a practical approach for examining gas flow behaviors in shale formation^8–10^. The MD method allows visualization of molecular movement and computation interactions among particles by determining the intermolecular and intramolecular interactions, accordingly providing a practical method for examining and clarifying gas adsorption and diffusion performances^11–13^.

Shale reservoirs generally comprise organic and inorganic substances, with the primary gas adsorption attributed to the organic matter. Moreover, the organic matter is fundamentally composed of kerogen^6,8, 14^. Due to its physical and chemical nature, type II-D kerogen, falling in the thermogenic gas window range, is usually utilized as the predominant unit in established shale models. Li and Sun investigated the CH_4_ and CO_2_ adsorption properties with various moisture contents in the type II-D kerogen matrix. They discovered that H_2_O molecules predominantly occupy superior adsorption sites on the shale surface and inside the shale matrix. As a result, water molecules drive CH_4_ and CO_2_ to reside in the bulk pore space, and H_2_O distribution in shale reservoirs restricts the CH_4_ and CO_2_ adsorption^6,7, 14^. Liu et al. probed the stability of the water bridge during the shale gas diffusion. They observed that CH_4_ merely diffuses at the interface of the water phase, whereas partial CO_2_ could penetrate the liquid water phase, owing to the high solubility of CO_2_ in water. High pressure restricted the diffusion process and enhanced CO_2_ solubility in the water bridge by strengthening the interactions between CO_2_ and H_2_O molecules^4^. Therefore, CO_2_ was a dominant factor influencing water bridge stability over CH_4_, due to the soluble process^4^. Tuan et al. observed that the water distribution in shale organic pores is essential for hydrocarbon adsorption and crucial in hydraulic fracture^15^. Tuan constructed three kerogen models by MD simulation and observed that the organic-pore systems exhibited mixed-wet characteristics, which might lead to a high potential for water entrapment and hinder gas adsorption and desorption processes^15^. Furthermore, another study by Tuan demonstrated that the contact angle and wettability of water on shale were influenced by gas injection pressure; the growing CO_2_ injection pressure increases the water contact angle and makes the water more hydrophobic on the organic surface, favoring gas recovery^16^. However, despite these findings, there still needs to be more understanding regarding the impact of water bridges on gas behavior and the additional ions in the water-bridge systems on efficient shale gas extraction and development.

This work uses MD simulation to construct a kerogen model representing the shale gas reservoir. Subsequently, the adsorption and density distribution profiles of CH_4_ and CO_2_ with a water bridge are investigated and examined within the established shale model. Furthermore, ions are introduced into the water system to mimic the mineralized formation water, enhancing the reliability and accuracy of simulation results for future gas extraction and recovery.

## Methodology

### Adsorbent model and construction

**Figure 1:**
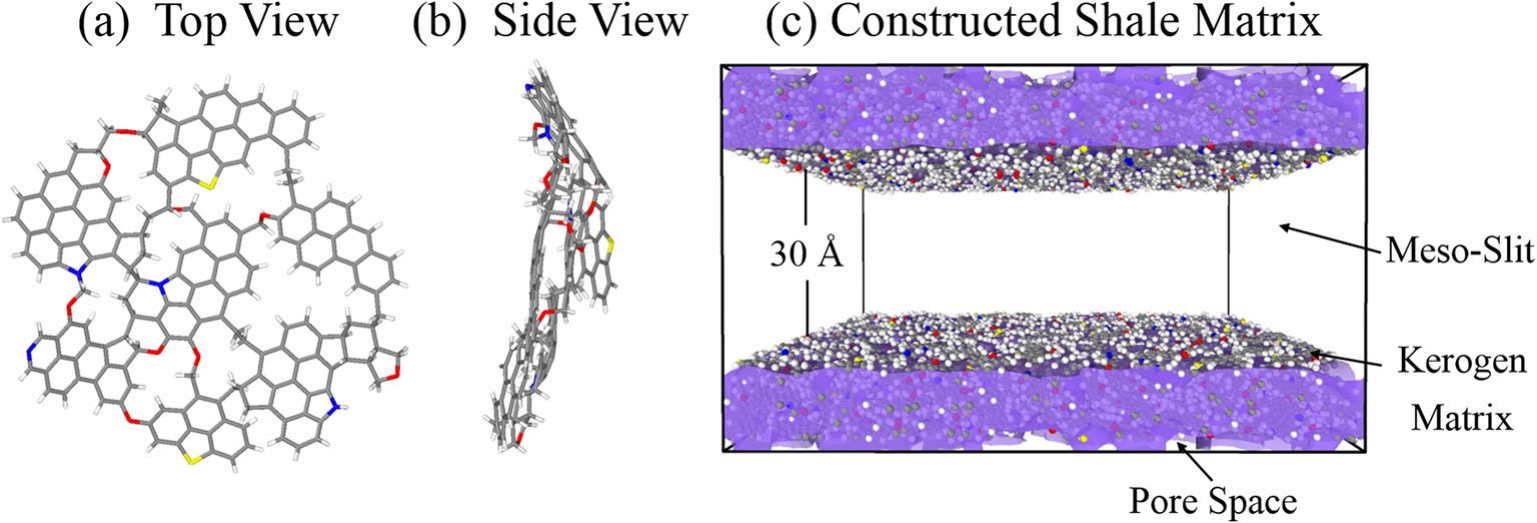
The (**a**) top view and (**b**) side view of atomic structure for the type II-D kerogen unit with chemical formula of C_175_H_102_N_4_O_9_S_2_, (**c**) the constructed shale matrix with meso-slit and nanopores rendered by the pore surface (purple color). (Atom representation: grey color for C, white color for H, red color for O, blue color for N, and yellow color for S.).

The type II-D kerogen unit at top and side views are depicted in Fig. 1a,b, and the unit is employed for establishing a realistic organic shale model, which ties the meso-slit and micropores characteristics in one, presenting the primary physical and chemical properties of shale gas-bearing reservoirs. The detailed physical and chemical information regarding type II-D kerogen is shown in Table 1. In order to generate this shale model, 100 units of C_175_H_102_N_4_O_9_S_2_ are employed to structure the organic matrix, as shown in Fig. 1c. The model generation and simulation processes, including gas adsorption determination and diffusion calculation, are conducted in the Large-scale Atomic/Molecular Massively Parallel Simulator (LAMMPS)^17^.

**Table 1:** Structural parameters of type ii-d kerogen^18^.

Model parameter	Value
H/C	0.58
O/C	0.051
N/C	0.023
S/C	0.011
Aromatic carbon (%)	79
Average number of carbons in each aromatic cluster	19.9
Fraction of aromatic carbons with attachments (sp^3^ C, N, S, O)	0.28
Protonated aromatic carbons (per 100 C)	25
Number of O in C–O (per 100 C)	5.1
Pyrrolic (mol % of N)	75
Pyridinic (mol % of N)	15
Aromatic sulfur (% of organic S)	100

The initial step was establishing the organic model by relaxing the single kerogen fragment at 338 K until the geometry was optimized. Subsequently, 100 units were placed in a 200^3^ Å^3^ box, with each region containing 50 units in the upper and lower regions to obtain the initial configuration of the shale matrix. During this step, two helium slabs are input into the system to maintain the height of the 30 Å meso-slit embedded in the shale model. The Constant Isobaric-Isothermic Ensemble (NPT) was then employed to achieve the shale system's final target temperature and pressure. NPT cycles were implemented with the pressure increases from 0.1 to 20 MPa while simultaneously controlling temperature drops from 1000 to 338 K. Each NPT cycle consisted of 2,000,000 steps to reach an equilibrium state. Following this, a Canonical Ensemble (NVT) was employed for another 2,000,000 steps to obtain the final stabilized configuration at 338 K and a pressure of 20 MPa. Therefore, the equilibrium density was determined as 1.36 g/cm^3^, lying in the range of 1.15–1.65 g/cm^3^ for shale kerogen density^19–21^. Moreover, the surface area was 570.45 m^2^/g, consistent with previous studies by Peng^22^ and shales in Eagle Fold Play and Marcellus Play^23^. The porosity calculated in the two pieces of software, namely, Ovito and PoreBlazer^24,25^, was determined to be 27.41%, falling in the range of 26–34% for the kerogen matrices^26,27^. This study combines the micropores and mesopores as a union to extract multi-scale physical characteristics of natural shale reservoirs, making the simulation environment more approach to the realistic geological condition and the simulation results more reliable.

### Adsorbate models

This work employs the Optimized Potentials for Liquid Simulations All-Atom (OPLS-AA) force field to describe CH_4_, which characterizes each atom thoroughly without sacrificing the intramolecular interactions to promote simulation efficiency, demonstrating a high accuracy in predicting hydrocarbon physical properties^28^. The elementary physical model (EPM2) force field is adopted to represent CO_2_. The extended simple point charge (SPC/E) model describes H_2_O molecules with charges assigned to each atom, ensuring precise results for further analysis. The consistent valence force field (CVFF) force field defines the kerogen matrix. Saline environments with a concentration of 3 mol/L NaCl are modeled using the clay force field (CLAYFF), incorporating Na^+^ and Cl^−^ ions to investigate their effect on gas adsorption and distribution behavior surrounding the water bridge. A generic force field, the DREIDING force field, is used to compute intramolecular interactions among the shale model, H_2_O molecules, ions, and gas particles. The detailed force field information is presented in Table S1 of the Supplementary information. Validation of the developed models can be found in Fig. S1 of the Supplementary information, which has successfully reproduced the macroscopic properties of fluids from the National Institute of Standards and Technology (NIST) database^5–7, 14^.

The DREIDING force field potential consists of two essentials, i.e., the bonding and non-bonding factors. The bonding part includes the bond stretch term (*E*_*b*_), the angle-bend term (*E*_*a*_), the torsion term (*E*_*t*_), the out-of-plane angle term (*E*_*o*_), and the cross-coupling term (*E*_*c*_), which are expressed as follows^29^,

1 $$E_{potential} = \, E_{bond} + \, E_{{non{ - }bonded}}$$

2 $$E_{bond} = \, E_{b} + \, E_{a} + \, E_{t} + \, E_{o} + \, E_{c}$$

The non-bonding part includes the van der Waals (vdW) interaction represented by the Lennard–Jones 12-6 potential, and the Coulombic potential, described as follows^29^,

3 $$E_{non - bonded} = 4\varepsilon_{XY} \left[ {\left( {\frac{{\sigma_{XY} }}{{r_{ij} }}} \right)^{12} - \left( {\frac{{\sigma_{XY} }}{{r_{ij} }}} \right)^{6} } \right] + k_{e} \frac{{q_{X} q_{Y} }}{{r_{ij} }}$$

where *ε*_*XY*_ stands for the Lennard–Jones potential well depth, and *σ*_*XY*_ represents the zero-potential distance. *r*_*ij*_ corresponds to the distance between particles of *i* and *j*, and *q* is the charge of particles. In addition, the cross term is computed by applying the Lorentz–Berthelot mixing rule as below,

3 $$\sigma_{ij} = \frac{1}{2} \left( {\sigma_{ii} + \sigma_{jj} } \right)$$

4 $$\varepsilon_{ij} = \sqrt {\varepsilon_{ii} \times \varepsilon_{jj} }$$

## Results and discussion

The density distributions of CH_4_ and CO_2_ are examined and discussed in this section to highlight water bridge’s impact and explore the water bridge’s stability from a visualized perspective, providing a molecular-scale analysis of the water bridge’s influence in shale reservoirs. In addition, the effects of temperature, pressure, and salinity on the adsorption and diffusion performances of CH_4_ and CO_2_ are investigated in this work.

### Density distribution of gas molecules

The gas density distribution is computed under various geological conditions, including the temperatures of 308 K, 338 K, and 368 K at pressures of 10 MPa and 20 MPa for pure CH_4_ and CO_2_. The amount of pre-absorbed water molecules needed to form a water bridge can be determined by the equation below,

5 $$\varpi = \frac{{N_{{H_{2} O}} *W_{{H_{2} O}} }}{{N_{{H_{2} O}} *W_{{H_{2} O}} + W_{skeleton} }}$$

where *N* represents the molecule number, and *W* represents the molar weight (g/mol). This work sets the moisture content as 11.15% of the matrix mass to enhance the water bridge effect. The quantity of H_2_O molecules was calculated as 1720. The introduced H_2_O molecules decreased the micropore volume by 44.96%, similar to that of a 44.1% reduction in a type II-D kerogen matrix with a 2 nm embedded slit by Stevens^19^, indicating the rationality and reliability of this study.

**Figure 2:**
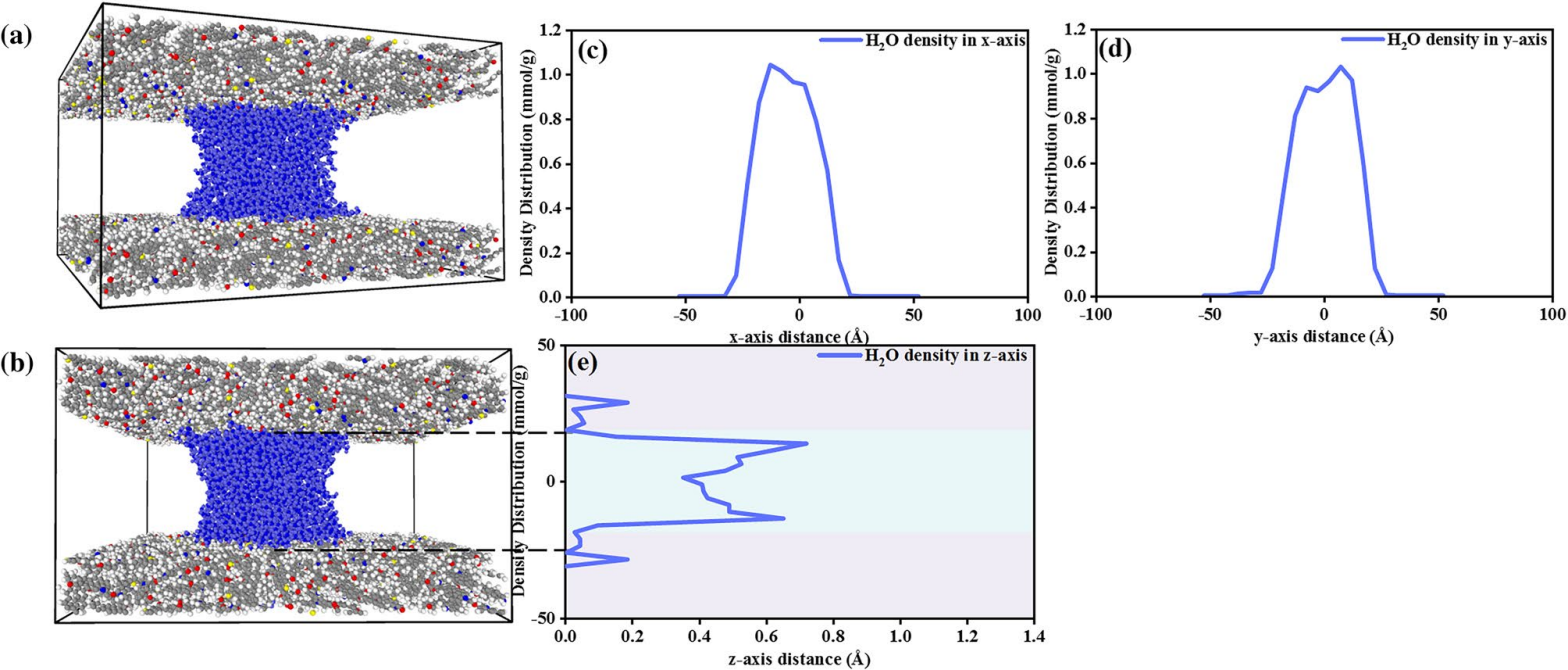
The (**a**) side view and (**b**) front view of water bridge atomic structure, (**c**–**e**) density distribution profiles of water bridge (H_2_O number of 1720), blueberry color for H atoms of H_2_O and navy color for O atoms of H_2_O.

The density profiles of the water bridge and gas molecules are determined by chunking the simulation system along three axes using the chunking command built into the LAMMPS software. The water density distribution profiles in three axes are plotted in Fig. 2 for better understanding. Based on the molecular distribution description in Fig. 2a,b, the water bridge distribution is in line with the observation in previous studies^30,31^. The increasing moisture content contributes to a water bridge formation between the shale slit. The density profile in Fig. 2c–e shows that the water bridge exhibits a narrow waist region, indicating weaker potential energy in this area, as seen in the center light blue area in the z direction, which lies between -12.5 Å and 12 Å.5 in Fig. 2e, presenting poor interactions between H_2_O molecules and the kerogen matrix. In contrast, the left light blue regions in Fig. 2e show a stronger connection between the water bridge and the kerogen surface, suggesting high potential energy and strengthened interaction between them, aligning with the findings by Sun and Liu^4,32^. This adsorption distribution suggests H_2_O molecules preferentially adsorb on the kerogen surface other than residing within the void slit in shale.

**Figure 3:**
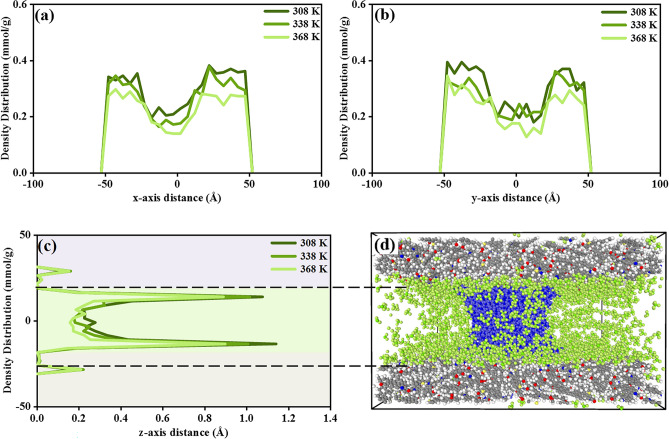
The (**a**–**c**) density distribution profiles of CH_4_ adsorption at 10 MPa under 308 K, 338 K, and 368 K, (**d**) front view of CH_4_ adsorption atomic structure with the presence of water bridge at 308 K and 10 MPa, lime color for C atoms of CH_4_ and green color for O atoms of CH_4_.

The density profiles of CH_4_ in three axes with the presence of water molecules at 10 MPa under temperatures of 308 K, 338 K, and 368 K are depicted in Fig. 3. The CH_4_ absolute adsorption under 10 MPa is 6.0 mmol/g, 5.4 mmol/g, and 4.6 mmol/g, respectively, 308 K, 338 K, and 368 K, with a water bridge. This adsorption pattern is in line with previous experimental studies by Billemont, and Ottiger^33–35^ conducted on Eagle Ford Play shale and Marcellus Play, of which are 5.8 mmol/g and 5.5 mmol/g at 318 K. The determined values in this work also algin with Sui, Heidari, and other simulation studies^36–38^ presented 5.2 mmol/g, 5.6 mmol/g, 6.1 mmol/g, and 5.5 mmol/g of absolute CH_4_ adsorption in shale gas reservoir, the adsorption deviations are negligible compared with this study. According to the density distribution in the z-axis, it is evident that CH_4_ exhibits a two-layer adsorption pattern in the shale model. The first adsorption peak shows up within the micropores featured kerogen matrix, while the secondary adsorption layer is observed near the kerogen surface inside the kerogen slit. The secondary adsorption layer is more noticeable than the first peak due to the micropores restraining the gas particles accumulation than that in meso-slit. Figure 3a–c demonstrate a decrease in CH_4_ adsorption across all directions with the increasing temperature and CH_4_ density distribution consistent with the outcomes by Huang^34^. Figure 3d presents an overview of the CH_4_ adsorption pattern with H_2_O. As the temperature increases, CH_4_ adsorption decreases insignificantly within the kerogen matrix but dramatically within the slit as the bulk phase as well as in the regions adjacent to the kerogen surface. This noticeable reduction in slit is attributed to the tremendous energy brought by the high temperature, allowing gas particles to be more energetic and wander around. The neglectable decrease within micropores of the kerogen matrix is attributed to the micropores having a more significant overlapping effect on gas adsorption, the increasing temperature enhances the collisions between gas molecules and the kerogen wall, thereby increasing the attachment potential, hence canceling the negative impact brought by the rising temperature to some extent.

**Figure 4:**
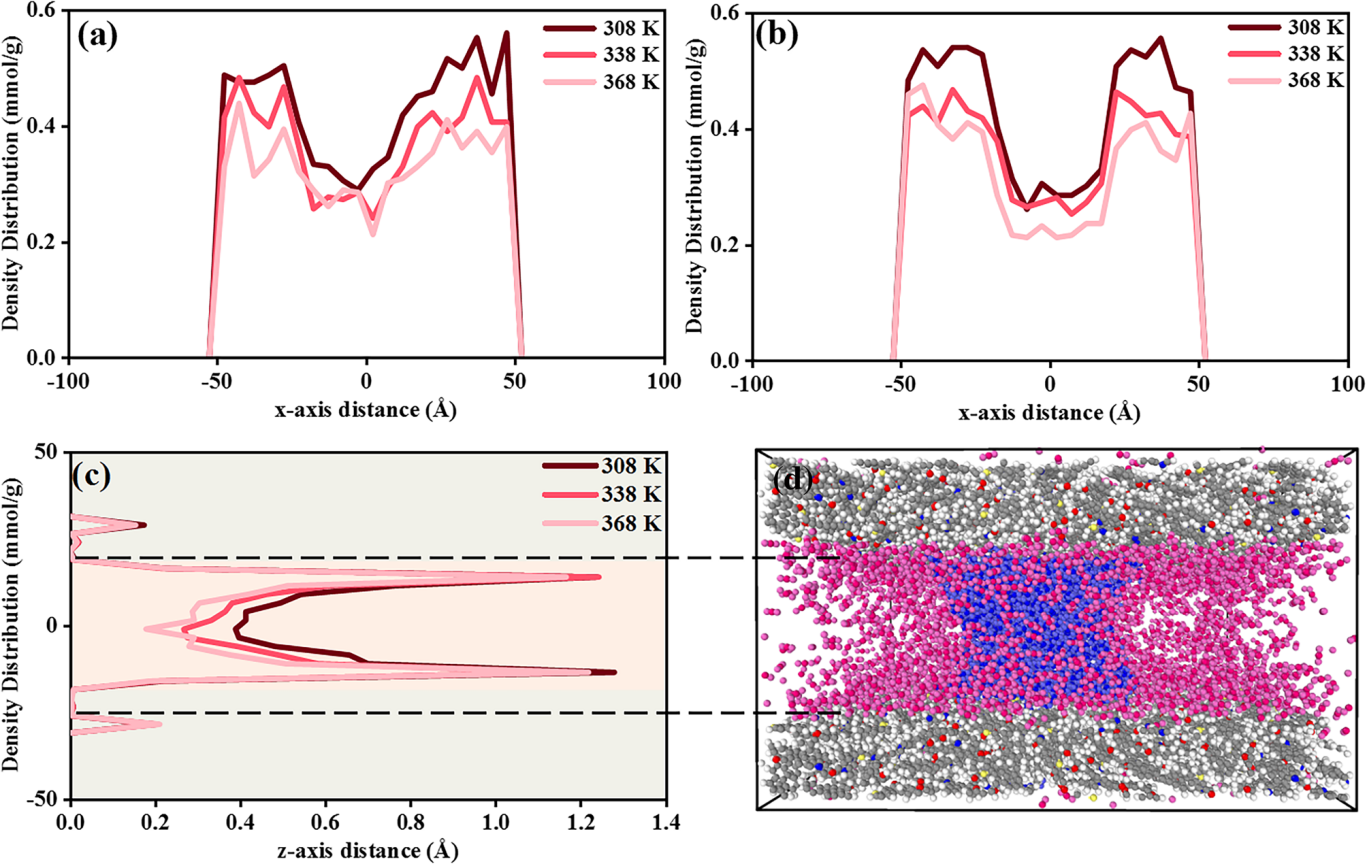
The (**a**–**c**) density distribution profiles of CO_2_ adsorption at 10 MPa under 308 K, 338 K, and 368 K, (**d**) front view of CO_2_ adsorption atomic structure with a water bridge at 308 K and 10 MPa, pink color for C atoms of CO_2_ and magenta color for O atoms of CO_2_.

The computed density profiles of CO_2_ in three axes with the water bridge at 10 MPa under temperatures of 308 K, 338 K, and 368 K are presented in Fig. 4. The CO_2_ absolute adsorption under 10 MPa is 8.6 mmol/g, 7.4 mmol/g, and 6.7 mmol/g at respectively 308 K, 338 K, and 368 K. This computed adsorption amount is in line with the observations by Raza, Ottiger, and other studies^35–37, 39, 40^, presenting 10.5 mmol/g in simulation, and 9.0 mmol/g by simulation under the same conditions. Figure 4a–c demonstrate a decreasing trend in CO_2_ adsorption amount with increasing temperature in all directions. Figure 4d presents an overview of CO_2_ adsorption behavior with H_2_O. Similar to CH_4_, the density profile of CO_2_ exhibits a two-layer adsorption structure in the direction of z-axis. The first adsorption is observed within the kerogen matrix, and the secondary adsorption layer lies adjacent to the kerogen surface. As the temperature grows, CO_2_ adsorption mildly shrinks in the kerogen matrix but significantly decreases within the slit as a bulk phase. Unlike CH_4_, there is no significant decrease in CO_2_ density next to the kerogen surface in the direction of z-axis, which is attributing to a stronger affinity between CO_2_ and shale than that of CH_4_ and shale.

**Figure 5:**
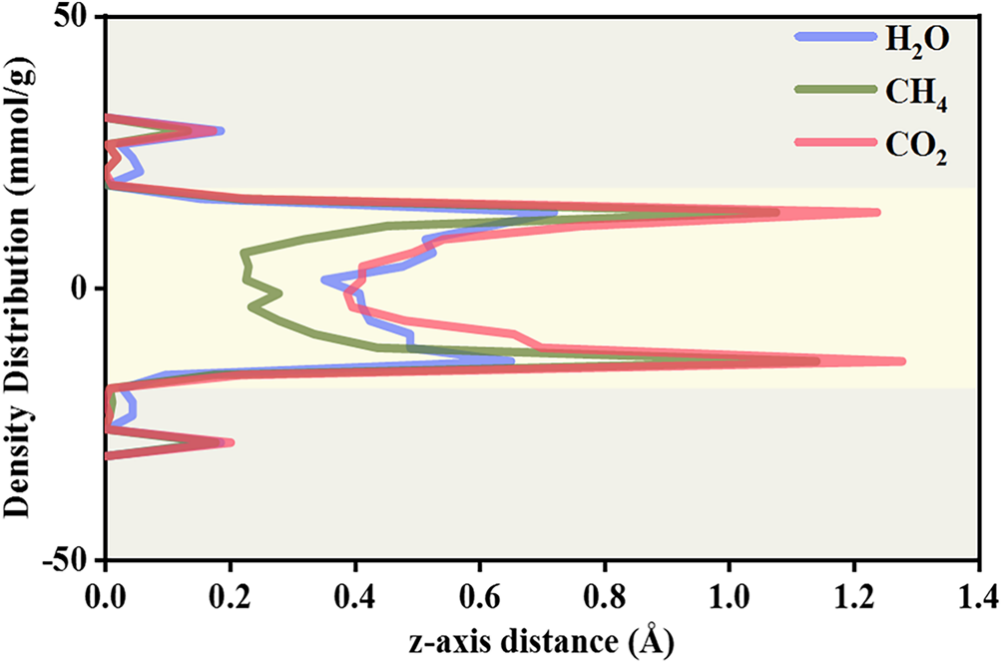
Density distribution of CH_4_, CO_2_, and H_2_O in direction of z-axis at 308 K and 10 MPa.

Figure 5 illustrates a water bridge presenting density profiles of CH_4_ and CO_2_ under 308 K and 10 MPa. Both CH_4_ and CO_2_ exhibit a two-layered adsorption shape within the kerogen matrix, showing the significant gas adsorption potential for the kerogen matrix; however, a higher CO_2_ adsorption amount within the kerogen matrix, suggesting more intense interactions between CO_2_ molecules and kerogen than that of CH_4_. Furthermore, CO_2_ shows higher density distribution in the bulk phase than CH_4_, indicating the preferential competitive adsorption performance of CO_2_ over CH_4_ within the matrix. CO_2_ has high sequestration potential in shale reservoirs, which suggests high capability in CO_2_ storage.

**Figure 6:**
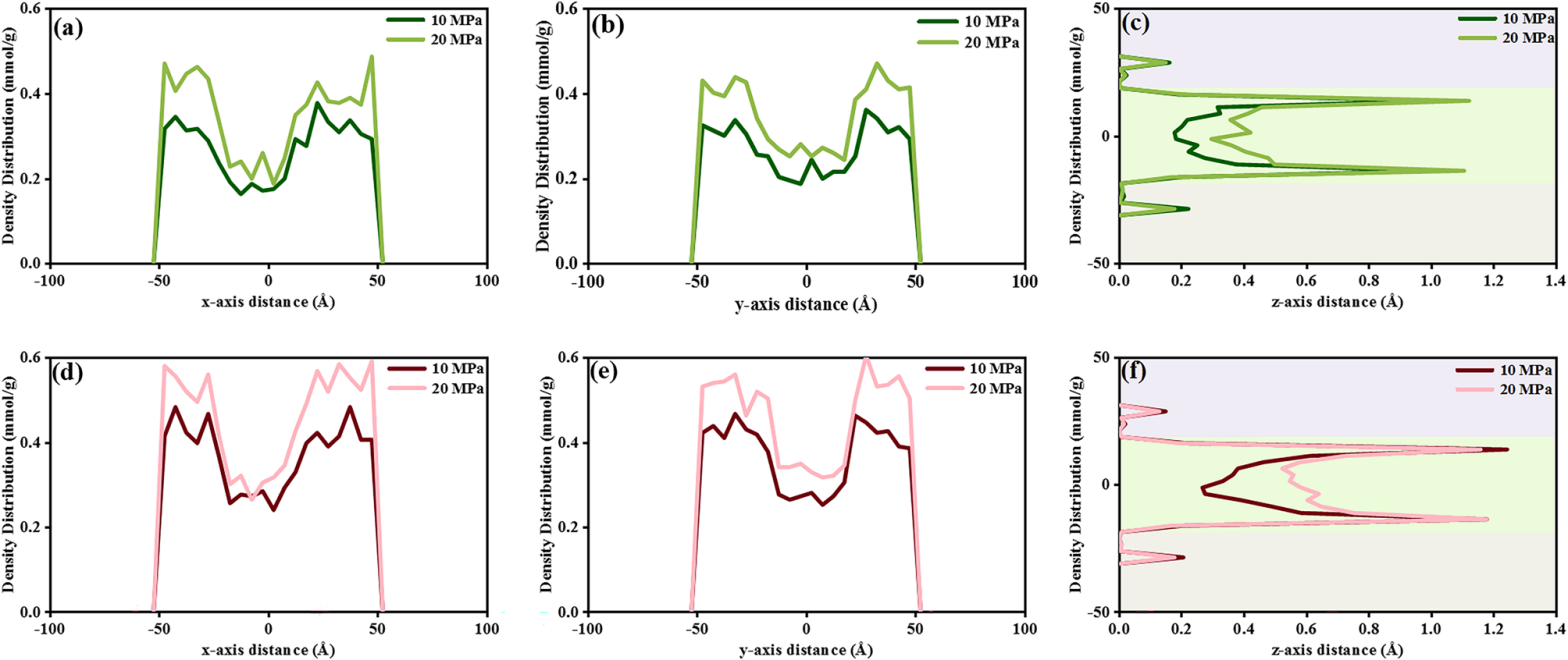
Density distribution in 3-axes of (**a**–**c**) CH_4_, and (**d**–**f**) CO_2_ at 338 K under 10 MPa and 20 MPa.

According to the analysis of the density distribution of CH_4_ and CO_2_ at 338 K under 10 MPa and 20 MPa, as shown in Fig. 6, the gas adsorption is enhanced in all directions with the rising pressure. The deviation is that CH_4_ has higher adsorption at high pressure in both the matrix surface region and in the kerogen slit as the bulk phase, as depicted in Fig. 6a–c. From Fig. 6d–f, CO_2_ density properties are similar to those of CH_4_ with pressure. On the contrary, the density profile of CO_2_ in the direction of the z-axis, as seen in Fig. 6f, shifts insignificantly on the matrix surface due to the almost fulfillment of the surface adsorption sites, but a noticeable increase in the matrix slit as the bulk phase.

### Saline effect in density distribution

Saline aquifers are abundant and geographically widespread worldwide^41,42^. Thereby, Na^+^ and Cl^−^ are introduced into the system to model the saline environment, as NaCl is considered the primary component of many formation brines^43^. The introduced H_2_O–NaCl system is usually employed to represent the fluid inclusions in various geological settings, such as hydrothermal mineral deposits, to quantify the salinity effect on gas adsorption inside the kerogen matrix.

**Figure 7:**
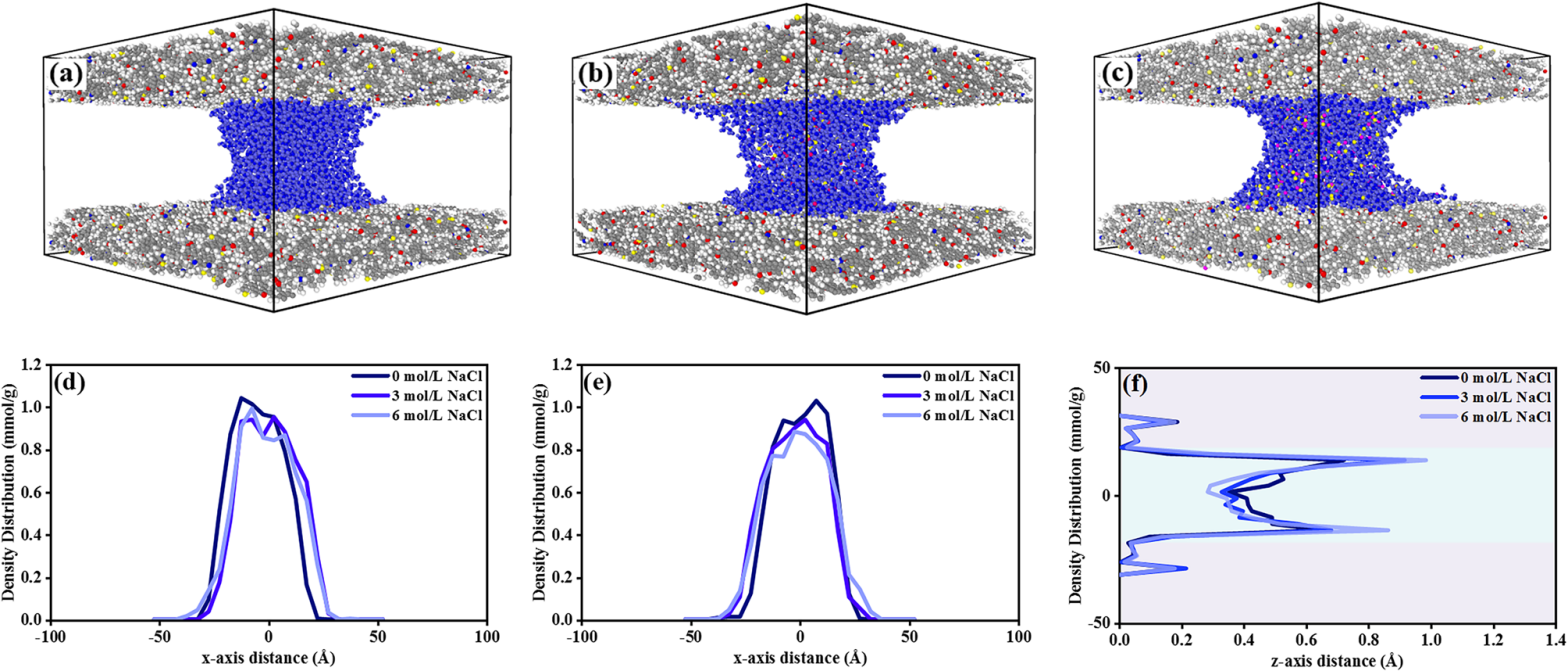
The (**a**–**c**) side view of pure, 3 mol/L, and 6 mol/L NaCl water bridge structure, (**d**–**f**) density distribution profiles of water bridge under different salinity in 3 axes, pink color for Na^+^ ions and yellow color for Cl^−^ ions.

The density profiles of H_2_O molecules with the presence of NaCl were computed in three directions, as depicted in Fig. 7. Figure 7a–c demonstrate the dynamic process of the water bridge shifting in the yz plane, which moves towards the center of the slit with the increasing NaCl concentration. In addition, the increasing concentration leads to a more uniform water distribution, which expands across the kerogen surface. This observation aligns with the simulation studies by Zhao, Xiong^44,45^, and the experimental works by Teklu and Wang^46,47^. This observation is evident in three axes, as shown in Fig. 7d,e. Furthermore, density distribution increases adjacent to the matrix surface, whereas, decreasing within the bulk phase with the concentration along the z-axis. This observation suggests that ions enhance the potential between H_2_O molecules and the matrix surface, resulting in preferential attachment of H_2_O molecules onto the kerogen surface. The scenario is attributed to negative and positive charges distributed on the shale surface; extra ions are introduced into the kerogen–water–gas system with the increasing concentration, H_2_O molecules enlarge along the shale surface, and the hydrophilicity of the kerogen surface increases with the growing concentration. This observation indicates an alternation in the shale surface wettability resulting from the contact angle reduction. Moreover, the amount of H_2_O molecules inside the matrix slits is reduced, implying ion weakens the potential energy at the water waist. In contrast, there is negligible H_2_O distribution variation within kerogen nanopores, owing to the adsorption sites within the matrix being almost filled with H_2_O molecules. The increasing concentration cannot further enhance H_2_O adsorption inside the nanopores of the matrix.

**Figure 8:**
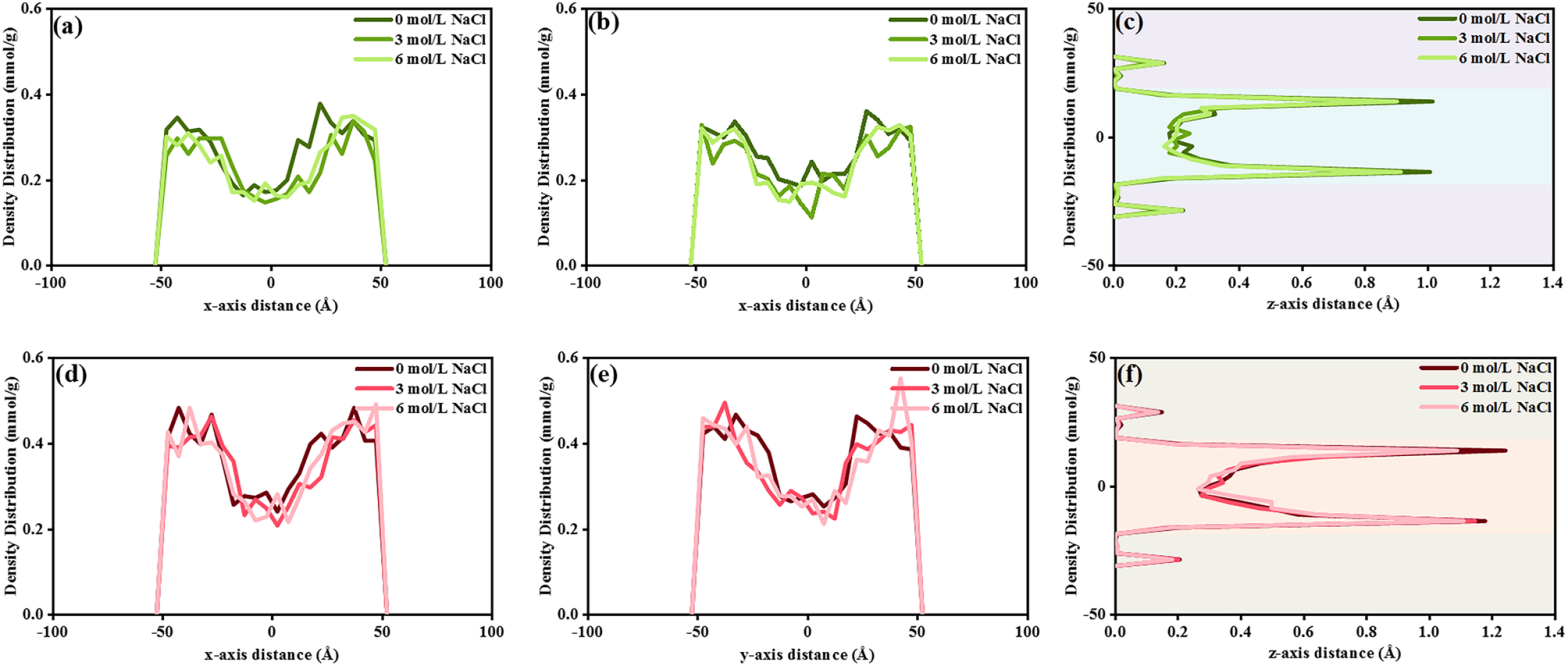
(**a**–**c**) CH_4_, and (**d**–**f**) CO_2_ density profiles in 3 axes under 0 mol/L, 3 mol/L, and 6 mol/L NaCl water bridge environment.

According to the density distributions of CH_4_ and CO_2_ in Fig. 8a–c, CH_4_ exhibits a decreasing trend at the matrix surface with concentration. This observation is in line with the experimental study by Zhang. Coal samples from the Baode block underwent a noticeable CH_4_ reduction with increasing salinity from 1000 to 2200 mg/L^48^. This CH_4_ reduction consists of the simulation outcomes on a type II-D kerogen matrix that experienced a NaCl encroachment from 2.5 to 6 mol/L by Zhou^49^. Figure 8a,b depict insignificant CH_4_ adsorption decreases at the boundaries of the x and y axes, whereas a particular reduction along the z-axis in Fig. 8c, attributed to the increased salinity promoting the interaction between H_2_O and kerogen, causes H_2_O to occupy the adsorption sites previously held by CH_4_. On the contrary, CO_2_ presents a distinct adsorption pattern, as depicted in Fig. 8d,e, CO_2_ demonstrates a slight adsorption increase on the kerogen surface with increasing salinity due to its favorable solubility in H_2_O, neutralizing the negative impact of H_2_O preferential adsorption. These observations provide evidence for the dynamic response of CO_2_ influence on H_2_O penetration into the kerogen matrix, suggesting that CO_2_ alters shale surface wettability.

### Gas diffusion behavior and mean square distance

Gas diffusion plays a crucial role during industrial gas flow, the organic nanopores restrain the gas diffusion process in shale, profoundly impacting both CH_4_ recovery and CO_2_ storage processes. The mean square distance (MSD) indicator is usually adopted to compute the diffusivity of gas transport and investigate the gas diffusion process. Previous studies^50–54^ have simplified this determination by considering *D*_*s*_ as the slope divided by six from linear regression analysis of MSD curves; however, this approach may lead to unexpected deviations in self-diffusivity results. This study combines the MSD and Einstein methods for a more precise self-diffusion coefficient estimation. The Einstein equation is expressed as follows^50^:

6 $$D_{s} = \frac{1}{6N}\mathop {\lim }\limits_{t \to \infty } \frac{d}{dt}\left\langle {\mathop \sum \limits_{k = 1}^{N} \left[ {r_{k} (t) - r_{k} (0)} \right]^{2} } \right\rangle$$

where *D*_*s*_ is the self-diffusion coefficient, representing the random motions or mixing of particles in the thermodynamic equilibrium.* N* is the gas molecular number, and *k* is the simulation time. *r*_*k*_(*t*) and *r*_*k*_(0) are the position vectors at *t* time and initial time. This section investigates the gas diffusion behavior for CH_4_ and CO_2_ in the water bridge system under 10 MPa and 20 MPa with and without ions.

**Figure 9:**
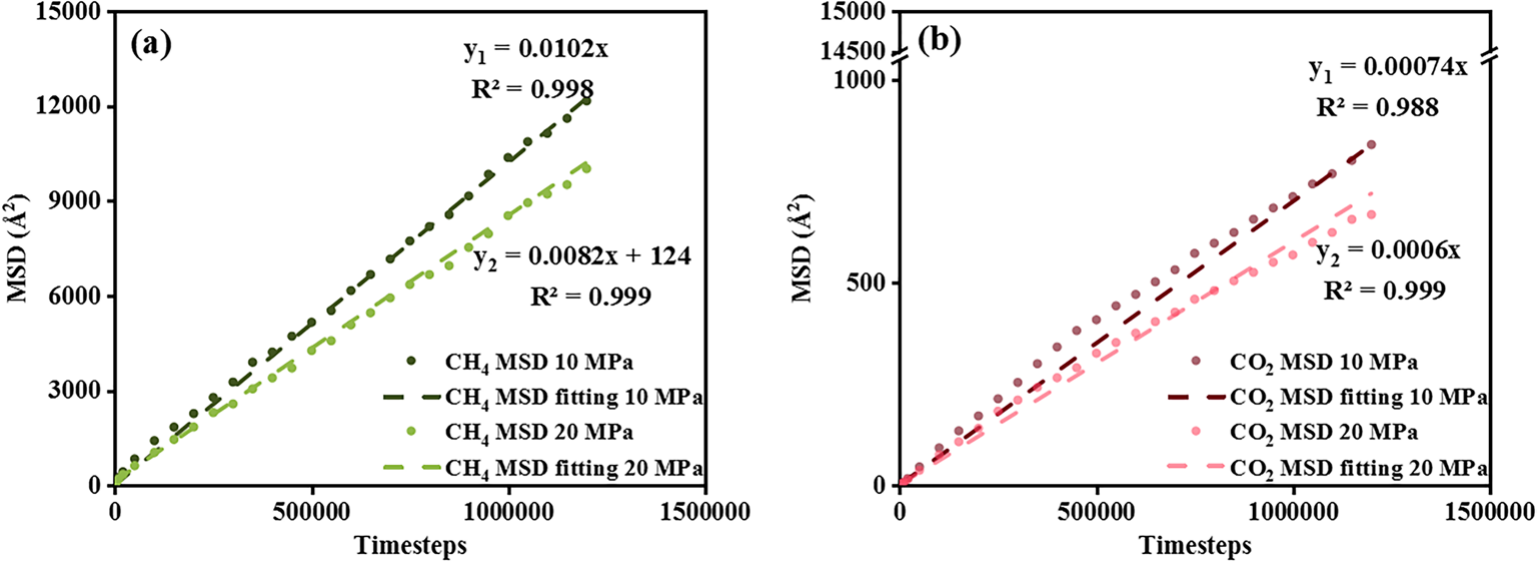
MSD of (**a**) CH_4_, and (**b**) CO_2_ under 338 K at 10 and 20 MPa.

Figure 9 depicts the MSD plots for CH_4_ and CO_2_. An initial unstable stage at the early timesteps is observed for both gases, particularly at low pressure. Then, the MSD profile stabilized at an equilibrium stage. A noticeable MSD reduction is observed at 20 MPa compared to 10 MPa for CH_4_ and CO_2_, indicating that high pressure restricts gas diffusion. CH_4_ in Fig. 9a presents a steeper MSD slope than CO_2_ in Fig. 9b, suggesting CO_2_ preferentially tends to attach to the adsorption sites, whereas CH_4_ predominantly resides within the slit as the bulk phase under the same condition. CO_2_ acts out a preferential adsorption behavior on shale over CH_4_, even with the presence of the water bridge.

**Figure 10:**
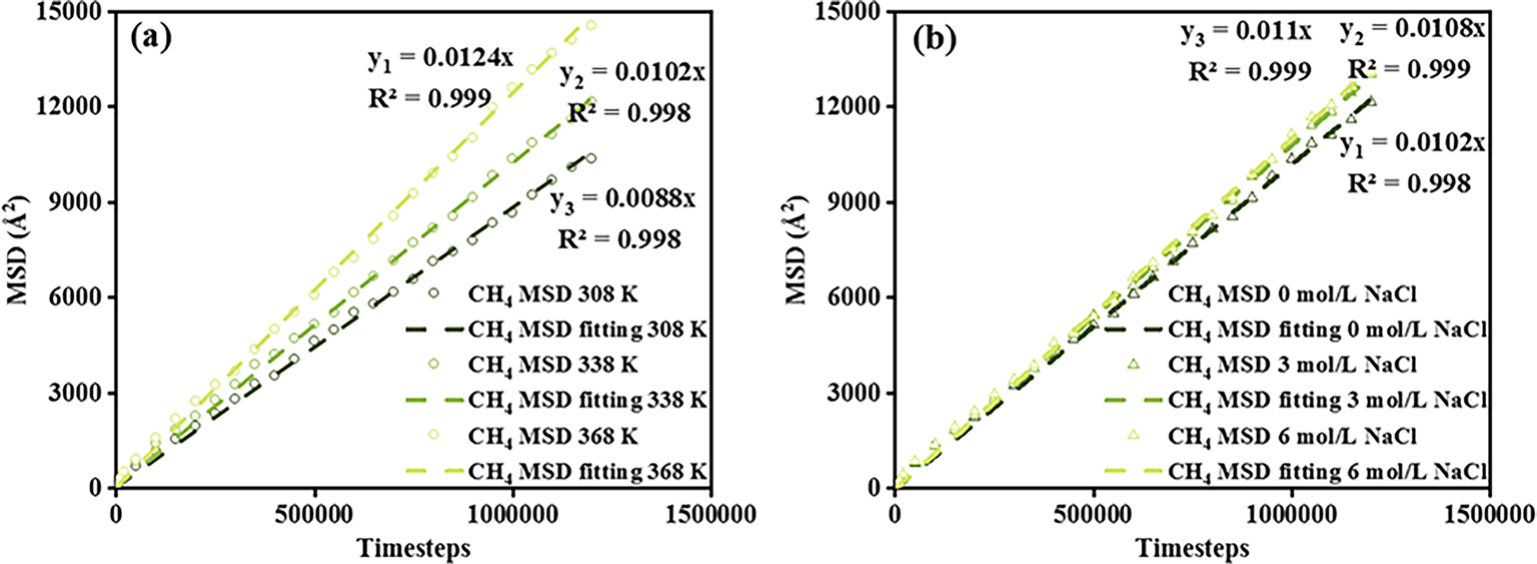
(**a**) MSD of CH_4_ under 10 MPa at 308 K, 338 K, and 368 K, (**b**) MSD of CH_4_ at 338 K with the presence of a water bridge of concentration of 0 mol/L, 3 mol/L, and 6 mol/L.

Figure 10a illustrates the MSD variation with the temperature conducted at 308 K, 338 K, and 368 K under 10 MPa. The MSD slope exhibits significant growth with increasing temperature, from 0.088 cm^2^/s at 308 K to 1.02 cm^2^/s at 338 K and further to 1.24 cm^2^/s at 368 K, which aligns the observation by Sun and Zhou^50,55^. This scenario is attributing to the high temperature providing more energy and facilitating the more intense particles’ movement transporting along the passing channel. Different NaCl concentrations are added into the water system to probe the encroachment effect on gas diffusion, the computed results of which are illustrated in Fig. 10b. As salinity increased, MSD increased a little from 1.02 cm^2^/s in pure water bridge to 1.08 cm^2^/s and 1.1 cm^2^/s at NaCl 3 mol/L and 6 mol/L concentrations, respectively. This enhancement can be attributed to the above-flattened water bridge attachment on the shale surface with increasing salinity, making a thinner waist and leaving more void space for CH_4_ to traverse.

**Figure 11:**
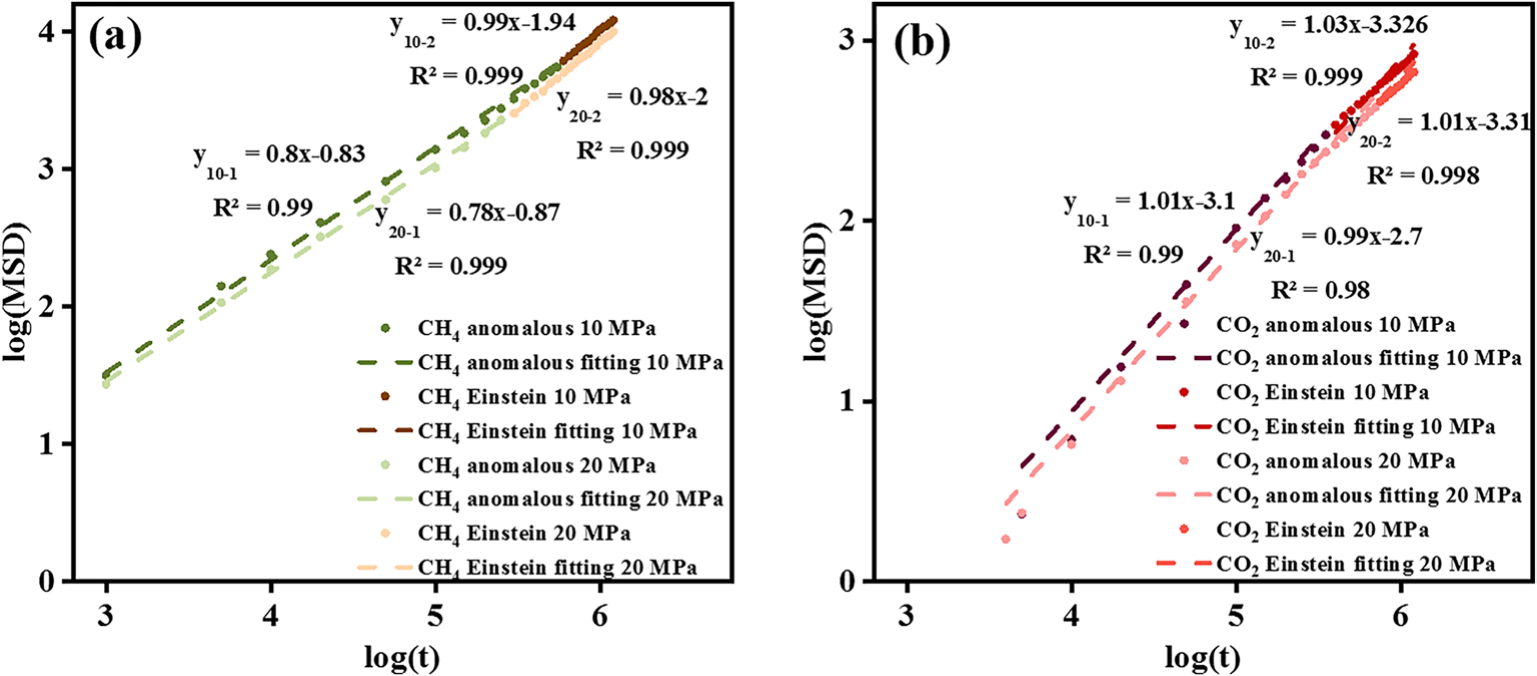
Self-diffusion of (**a**) CH_4_, and (**b**) CO_2_ under 338 K at 10 MPa and 20 MPa.

The Einstein determination comprises two stages, the anomalous diffusion represents the undersaturated gas state where molecules under full adsorption attempt to occupy vacant sites. In contrast, Einstein diffusion characterizes the saturated gas state where molecules attach to available sites and achieve a stable diffusion state. Figure 11 yields consistent findings that the increasing pressure restricts the self-diffusion coefficient in anomalous and Einstein stages for CH_4_ and CO_2_. Under saturation conditions, the CH_4_ self-diffusivity slope in Fig. 11 is 0.99 × 10^−9^ m^2^/s (orange line), which aligns with the previous results of 0.97 × 10^−9^ m^2^/s by Dawass et al.^56–59^. The thermodynamic properties of CO_2_ are illustrated in Fig. 11b, exhibiting a higher self-diffusion coefficient than CH_4_, indicating that the smaller aerodynamic diameter holder CO_2_ passes through kerogen meso-slit faster to get saturated conditions. The self-diffusion coefficient curves at two Einstein stages at 20 MPa are below 10 MPa, suggesting that increasing pressure makes gas diffusion challenging.

**Figure 12:**
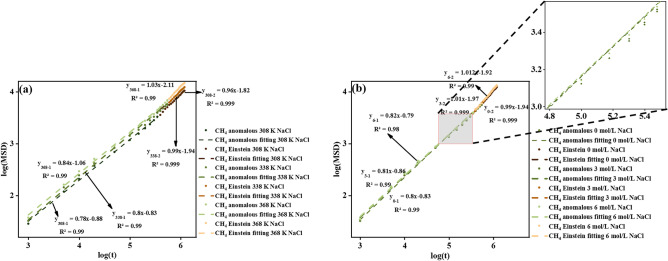
Self-diffusion coefficients of (**a**) CH_4_ under 10 MPa at 308 K, 338 K, and 368 K, (**b**) CH_4_ at 338 K with the presence of a water bridge of concentration of 0 mol/L, 3 mol/L, and 6 mol/L.

Figure 12a demonstrates an increase in self-diffusion coefficient with rising temperature, consistent with the above-mentioned observations depicted in Fig. 10a. During the anomalous stage, a more pronounced deviation in self-diffusion coefficient as CH_4_ molecules tend to occupy available adsorption sites. Followed by a reduced deviation scale during the Einstein stage, CH_4_ almost saturated and travels as the bulk phase. In addition, CH_4_ shows minor shifting with ions despite the concentration increases from 0 to 6 mol/L in Fig. 12b, owing to the non-polarity characteristic of CH_4_ weakening the interactions between CH_4_ and kerogen. However, the more flattened water bridges distribution on the kerogen surface enables more space for CH_4_ molecules to wander inside.

## Conclusions

This work has novelty investigated the gas adsorption and transportation behavior in shale gas reservoir with formation water, which has usually been neglected or overlooked in previous studies. Notably, this work induced various salinity approaches to a more reliable subsurface environment. Based on the discussion in section “Results and discussion”, the water bridge formed inside the shale reservoir negatively works on gas distribution, particularly water-contained ions inside the underground system, further impeding the gas transport and adsorption pattern. This study has probed and discussed gas adsorption characteristics, distribution profile, and diffusion behavior under 308 K, 338 K, and 368 K at 10 MPa and 20 MPa with a water bridge. Furthermore, 3–6 mol/L NaCl are added into the system, the effect of the ion on the water bridge pattern on the shale surface and interaction with gas particles. This approach is more suitable for modeling underground environments and can enhance the realism of gas exploration and extraction. The conclusions are drawn as follows,

1. CH_4_ and CO_2_ adsorption on the kerogen and slit decreases with the presence of a water bridge, owing to the preferential interaction between H_2_O and kerogen enhancing the H_2_O capture of the available adsorption sites, thereby leaving CH_4_ and CO_2_ less favorable adsorption environment.
2. The increasing salinity of NaCl weakens CH_4_ and CO_2_ adsorption on the kerogen surface added into the water system, attributed to the ions enhancing the interaction between H_2_O and kerogen, thereby reducing available adsorption sites for gas particles and resulting in a diminished adsorption pattern.
3. The diffusivities of CH_4_ and CO_2_ are constrained by the increasing pressure in this work, attributed to the external force that restricts the movement of gas particles. Conversely, increasing temperature enhances gas diffusivity by providing more energy. Moreover, the growing salinity has an insignificant influence on gas diffusivity.

### Supplementary Information

Supplementary Information.

## Data Availability

The datasets used and/or analysed during the current study available from the corresponding author on reasonable request.

## Abbreviations

CCUS: Carbon capture, utilization, and storage
MD: Molecular dynamics
LAMMPS: Large-scale atomic/molecular massively parallel simulator
NPT: Constant isobaric-isothermic ensemble
NVT: Canonical ensemble
OPLS-AA: Optimized potentials for liquid simulations all-atom
EPM2: Elementary physical model
SPC/E: Extended simple point charge
CVFF: Consistent valence force field
CLAYFF: Clay force field
NIST: National Institute of Standards and Technology
vdW: Van der Waals forces
MSD: Mean square distance
