# Supplementary material for: The role of water bridge on gas adsorption and transportation mechanisms in organic shale
Source: Sci Rep. 2024 Jul 1;14:15008. doi: 10.1038/s41598-024-66055-4 (PMC11217271; doi:10.1038/s41598-024-66055-4)
Supplement: Supplementary file 1 — Supplementary Information. [file 41598_2024_66055_MOESM1_ESM.pdf]

Supporting Information for

The Role of Water Bridge on Gas Adsorption and Transportation Mechanisms in Organic Shale

Binhui Li [a,b], Yong Liu [a,b], Yubo Lan [a,b], Jiawei Li [a,b,c,*], Yue Lang [b], Sheikh S Rahman [c]

[a] *State Key Laboratory of Continental Shale Oil, Daqing 163712, China*

[b] *Daqing Oilfield Exploration and Development Research Institute, Daqing, Heilongjiang, 163712, China*

[c] *School of Minerals and Energy Resources Engineering, UNSW, Sydney 2052, Australia*

* Corresponding author at: State Key Laboratory of Continental Shale Oil, Daqing 163712, China

Email address: z5056275@zmail.unsw.edu.au (J. Li)

Contents of this file:

1. Tables S1
2. Figures S1
3. SI References

Table S1. The force field parameters for CH_4, CO_2, H_2O and NaCl.

Molecule	Atom	ε/k_b (K)	σ (Å)	q (e)	Reference
CH_4	C	33.212	3.5	-0.24	1,2
	H	15.097	2.5	0.06	
CO_2	C	28.129	2.757	+0.6512	3,4
	O	80.507	3.033	−0.3256	
H_2O	H	0	0	0.4238	5,6
	O	78.197	3.166	-0.8476	
NaCl	Na	65.52	2.3502	+1.0	7
	Cl	50.36	4.4	-1.0	

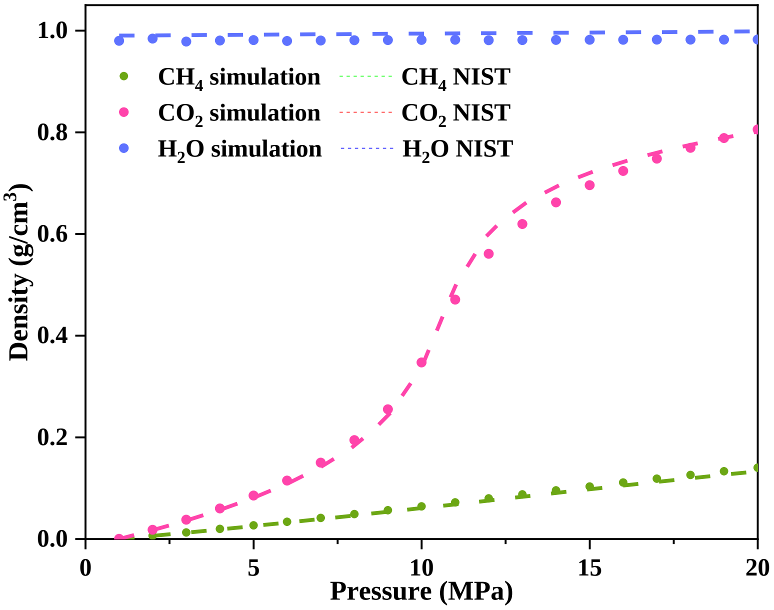

Figure S1. Fluid bulk density validations by comparing the simulated results with the NIST data at 318.15 K from 0.1 to 20 MPa.

References

1. Jorgensen, William, L., Maxwell, David, S. Development and testing of the OPLS all-atom force field on conformational energetics and Properties of Organic Liquids. *Journal of the American Chemical Society.* 1996;118(45):11225-11236.
2. Chang J, Sandler SI. Interatomic Lennard-Jones potentials of linear and branched alkanes calibrated by Gibbs ensemble simulations for vapor-liquid equilibria. *The Journal of Chemical Physics.* 2004;121(15):7474-7483.
3. Aimoli CG, Maginn EJ, Abreu CR. Force field comparison and thermodynamic property

calculation of supercritical CO2 and CH4 using molecular dynamics simulations. *Fluid Phase Equilibria.* 2014;368:80-90.
4. Harris JG, Yung KH. Carbon Dioxide's Liquid-Vapor Coexistence Curve And Critical Properties as Predicted by a Simple Molecular Model. *The Journal of Physical Chemistry.* 1995;99(31):12021-12024.
5. Berendsen HJC, Grigera JR, Straatsma TP. The missing term in effective pair potentials. *The Journal of Physical Chemistry.* 1987;91(24):6269-6271.
6. Mark P, Nilsson L. Structure and Dynamics of the TIP3P, SPC, and SPC/E Water Models at 298 K. *The Journal of Physical Chemistry A.* 2001;105(43):9954-9960.
7. Cygan RT, Liang J-J, Kalinichev AG. Molecular Models of Hydroxide, Oxyhydroxide, and Clay Phases and the Development of a General Force Field. *The Journal of Physical Chemistry B.* 2004;108(4):1255-1266.
